# Metabolic Syndrome and Dementia Risk in Older Adults: A Narrative Review

**DOI:** 10.1002/kjm2.70276

**Published:** 2026-08-19

**Authors:** Hyojin Park, Mallika Chuansangeam, Yuan‐Han Yang

**Affiliations:** ^1^ Department of Family Medicine Hallym University Kangnam Sacred Heart Hospital Seoul Republic of Korea; ^2^ Division of Geriatric Medicine, Department of Internal Medicine, Faculty of Medicine Thammasat University Pathum Thani Thailand; ^3^ Department of Neurology Kaohsiung Medical University Gangshan Hospital, Kaohsiung Medical University Kaohsiung Taiwan; ^4^ Neuroscience Research Center Kaohsiung Medical University Kaohsiung Taiwan; ^5^ School of Post‐Baccalaureate Medicine, College of Medicine, Kaohsiung Medical University Kaohsiung Taiwan; ^6^ Department of Neurology Kaohsiung Medical University Hospital, Kaohsiung Medical University Kaohsiung Taiwan

**Keywords:** Alzheimer disease, cognitive dysfunction, dementia, metabolic syndrome, vascular dementia

## Abstract

Dementia is a major global health issue in aging societies. Metabolic syndrome (MetS), a cluster of metabolic abnormalities, has been proposed as a modifiable risk factor for dementia, although existing evidence remains inconsistent. This review examined recent studies on the association between MetS and dementia risk in older adults. We searched PubMed for original studies published between January 1, 2014, and July 15, 2024, using the keywords “metabolic syndrome” and “dementia.” Fourteen studies met the inclusion criteria: five retrospective cohort studies, two prospective cohort studies, four cross‐sectional studies, two bioinformatics studies, and one meta‐analysis. Overall, MetS was associated with adverse cognitive outcomes, with relatively more consistent evidence for vascular dementia and progression from mild cognitive impairment to dementia than for Alzheimer disease. Hypertension, abdominal obesity, and low high‐density lipoprotein cholesterol were associated with adverse cognitive outcomes in some studies, although component‐specific findings varied across studies. Temporal patterns of MetS also appeared to be relevant, with some studies reporting higher dementia risk in individuals with worsening or persistent metabolic burden, although findings after MetS resolution were not consistent. Several studies also reported deficits in executive, attention, and visuospatial function. Bioinformatics studies suggested possible genetic links, including overlap with apolipoprotein E‐related pathways, although evidence remains limited. MetS may represent a clinically relevant risk marker for adverse cognitive outcomes in older adults, although its association varies according to MetS definition, study population, and cognitive outcome. Further large‐scale studies are needed to clarify the underlying mechanisms and subtype‐specific associations.

## 1 Introduction

As the global population continues to age, the prevalence of dementia—a leading cause of cognitive decline—is rising rapidly. It is estimated that the number of dementia patients worldwide will increase by 166% from approximately 57.4 million in 2019 to 152.8 million in 2050 [1]. In South Korea, the prevalence of dementia in the elderly over 65 years of age is 8%–13%, and this rate approximately doubles with every 5‐year increase in age after 65 [2]. The social and economic burden is expected to increase rapidly with the increasing prevalence of dementia, prompting active research into various risk factors [3].

Among these risk factors, metabolic syndrome (MetS) has attracted attention, particularly, as its prevalence continues to rise due to modern lifestyle changes, including population aging, urbanization, reduced physical activity, and high‐calorie diets [1, 4]. In South Korea, the prevalence of MetS in adults is estimated at 24.9%, and the prevalence in the elderly over 65 years of age was confirmed to be 47.3%, much higher than the overall prevalence in adults. Notably, the highest prevalence is observed in individuals in their 70s, at 52.1% [5].

MetS is defined by the co‐occurrence of central obesity, hypertension, hyperglycemia, hypertriglyceridemia, and low high‐density lipoprotein cholesterol (HDL‐C). Each MetS factor can increase the risk of cardio‐cerebrovascular disease, making MetS a major global public health concern [6]. In older adults, MetS may also be associated with functional impairment, including slower gait speed and declines in physical and cognitive function [7]. As a result, an increasing number of studies have explored the impact of MetS on cognitive function and dementia [8, 9].

Mechanistically, MetS may lead to vascular dementia (VaD) through hypertension and arteriosclerosis, which compromise cerebral blood vessels and reduce cerebral blood flow [10]. Moreover, MetS may elevate the risk of Alzheimer disease (AD) by promoting β‐amyloid accumulation, insulin resistance, and chronic low‐grade inflammation [11]. Brain atrophy and functional changes induced by MetS are also suggested to contribute to neurodegeneration and cognitive decline [12].

However, despite the growing body of literature, findings on the association between MetS and dementia remain inconsistent, potentially due to heterogeneity in study design and populations [13]. Therefore, in this narrative review, we aim to provide a comprehensive summary of the existing evidence on the relationship between MetS and dementia risk, and to clarify the significance of MetS as a potentially modifiable risk factor for dementia (Table 1).

**TABLE 1:** Overview of included studies on the relationship between metabolic syndrome and risk of dementia.

References	Study design	Study sample	MetS definition or metabolic variables examined	Dementia type	Key findings
Li et al. [14]	Bioinformatics	Genes from National Center for Biotechnology Information (NCBI) Gene Expression Omnibus (GEO) database	MetS‐related genes; no clinical MetS definition applied	AD	Identified eight genes (e.g., ARHGAP4, SNRPG) common to AD and MetS; immune cell infiltration observed; The immune‐related gene SNRPG linked to glucose metabolism.
Pillai et al. [15]	Retrospective cohort	106 MCI, 50 dementia patients (age 55–90)	Plasma and CSF ApoA1, plasma TG/HDL‐C ratio, BBB biomarkers, and inflammatory biomarkers; no clinical MetS definition applied	AD	Elevated plasma TG/HDL‐C and ApoA1 associated with worse outcomes; higher CSF ApoA1 linked to slower MMSE decline in MCI.
Atti et al. [13]	Meta‐analysis	18,313 adults (cognitively normal or with MCI, aged > 40)	MetS; diagnostic criteria varied across the included studies	All‐cause dementia, AD, VaD	No pooled association with dementia or AD; MetS increased risk of VaD and MCI progression to dementia.
Ng et al. [16]	Prospective cohort	1519 cognitively normal adults (aged > 55)	MetS (modified NCEP ATP III)	MCI, dementia progression	MetS, central obesity, DM, dyslipidemia, and ≥ 3 components increased risk of MCI and progression to dementia.
Zhang et al. [17]	Bioinformatics	173 genes from DisGeNET database v4.0 discovery platform	Genes related to MetS, dementia, or DM; no clinical MetS definition applied	All‐cause dementia	*APOE, APP, PARK2*, and other genes identified as common nodes; APOE modestly associated with dementia and metabolic complications.
Viticchi et al. [18]	Cross‐sectional	162 AD patients (mean age 72.5 years)	MetS (NCEP‐ATP III)	AD	AD patients with MetS had lower BHI and MMSE scores than those without MetS.
Fan et al. [19]	Retrospective cohort	3458 adults (age 40–80)	MetS (modified NCEP ATP III)	All‐cause dementia	Worsened MetS increased dementia risk (HR 2.22); no significant risk with persistent or improved MetS. Similar results in ≥ 65 years.
Exalto et al. [20]	Prospective cohort	86 patients (MMSE > 22, mean age 66.7 years)	MetS (NCEP‐ATP III)	All‐cause dementia	MetS associated with poorer performance in executive function, attention/processing speed, and visuoconstruction; no difference in dementia conversion.
Lee et al. [21]	Retrospective cohort	12,296,863 adults (aged > 50, dementia‐free)	Metabolic health defined using NCEP ATP III components; obesity defined as BMI ≥ 25 kg/m^2^	All‐cause dementia, AD, VaD	Metabolically healthy obesity associated with lower risk of dementia and AD, but not VaD; other MetS components increased risk, especially for VaD.
Hishikawa et al. [22]	Retrospective cohort	570 ad patients (mean age 79.8 years)	MetS (Japanese diagnostic criteria based on waist circumference and ≥ 2 vascular risk factors); HOMA‐IR and RHI additionally assessed	AD	AD patients with MetS had worse affective symptoms, insulin resistance, and vascular endothelial dysfunction; cognitive scores were lower but not significantly different.
Marseglia et al. [14]	Cross‐sectional	1131 adults (mean age 70 years, dementia‐free)	MetS (IDF)	All‐cause dementia	MetS associated with worse attention/processing speed, executive function, and verbal fluency; effects modified by APOE‐ε4, education, and CVD.
Wu et al. and Chen [23]	Retrospective cohort	5693 adults from Taiwan Biobank (age 30–70)	MetS (modified NCEP ATP III criteria using population‐specific waist circumference cutoffs)	All‐cause dementia	Hypertension, abdominal obesity, and low HDL‐C linked to poorer MMSE orientation, language, and visuospatial scores; persistent MetS increased long‐term risk.
Deng et al. [24]	Cross‐sectional	1781 adults (aged > 60 years, China)	Individual metabolic and lifestyle risk factors assessed by questionnaire; no clinical MetS definition applied	All‐cause dementia	Risk factors: rural residence, older age, single status, obesity, HTN, CAD, depression. Protective factors: smoking, freshwater fish, exercise, housework, outdoor/social activities.
Kim et al. [25]	Cross‐sectional	19,935 adults (aged > 60 years, Korea)	Individual metabolic risk factors, including obesity, hypertension, DM, and stroke; no clinical MetS definition applied	All‐cause dementia	Stroke, HTN, and DM were key risk factors; exercise and alcohol consumption associated with lower dementia risk, with sex‐specific differences.

Abbreviations: AD, Alzheimer disease; ApoA1, apolipoprotein A1; BBB, blood–brain barrier; BHI, breath‐holding index; CVD, cardiovascular disease.DM, diabetes mellitus; HOMA‐IR, homeostasis model assessment of insulin resistance; IDF, International Diabetes Federation; MCI, mild cognitive impairment; MetS, metabolic syndrome; RHI, reactive hyperemia index; TG/HDL‐C, triglyceride‐to‐high‐density lipoprotein cholesterol ratio; VaD, vascular dementia.

## 2 Methods

### 2.1 Search Strategy

We searched PubMed for original articles using the keywords “metabolic syndrome” and “dementia.” Studies published between January 1, 2014, and July 15, 2024, were included. Only original articles published in English were considered. Studies involving only human participants were included, while animal or in vitro studies were excluded. Age filters of “65 years and older” and “80 years and older” were applied to focus on older populations. Studies were eligible if they included participants aged ≥ 65 years, even when the overall study population was broader. The reference lists of key publications were also screened to identify additional eligible studies. Eligible study designs included original prospective, retrospective, and cross‐sectional studies.

### 2.2 Selection Criteria

A total of 29 articles were initially identified through the PubMed search. After screening titles and abstracts, eight articles were excluded. Five additional articles were excluded because they were reviews or editorials rather than original research. One article involving in vitro research and one retracted article were also excluded.

**FIGURE 1:**
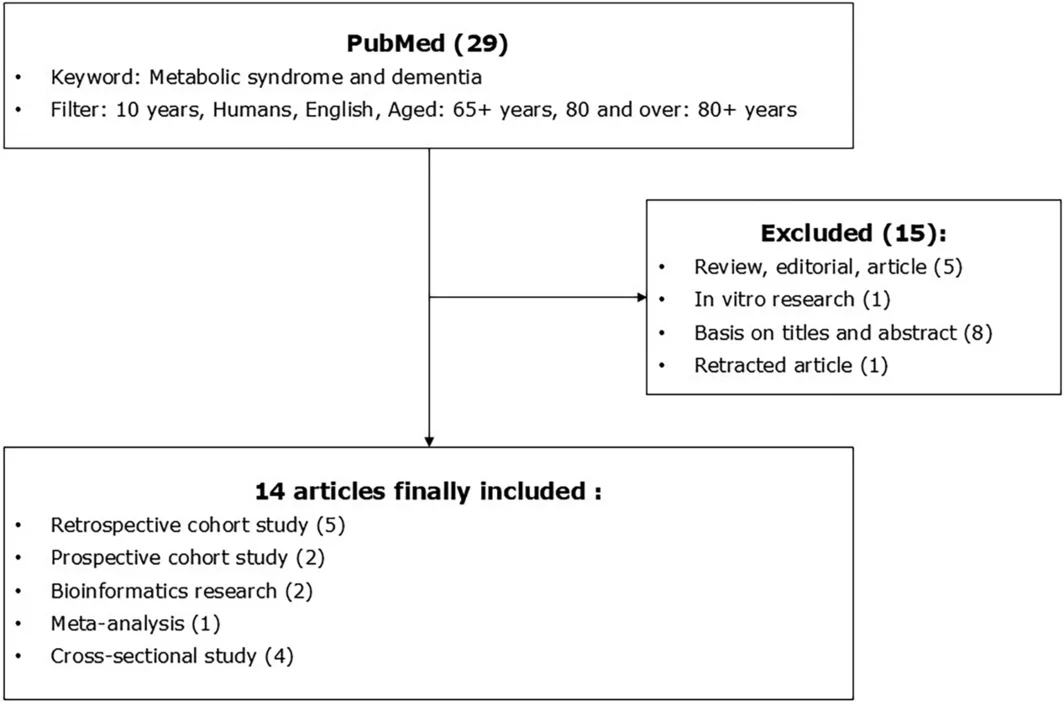
Flow diagram of included studies.

Ultimately, 14 articles met the inclusion criteria and were included in the final analysis. The final selection consisted of five retrospective cohort studies, two prospective cohort studies, four cross‐sectional studies, two bioinformatics studies, and one meta‐analysis. The study selection process is summarized in Figure 1.

### 2.3 Quality Assessment

This review was conducted as a narrative review; however, a structured methodological quality assessment of the included studies was performed to enhance transparency. We applied the Newcastle–Ottawa Scale for cohort studies, the Joanna Briggs Institute Critical Appraisal Checklist for cross‐sectional studies, and A Measurement Tool to Assess Systematic Reviews 2 for the included meta‐analyses. For bioinformatics‐based studies, no validated risk‐of‐bias tools were available; therefore, methodological rigor and inherent limitations were assessed qualitatively. The results of these assessments are shown in Table S1.

## 3 Results

### 3.1 MetS and the Risk of Dementia

We categorized the studies based on whether the study population included individuals with pre‐existing dementia.

#### 3.1.1 Studies Involving Dementia‐Free Populations

Atti et al. [13] conducted a meta‐analysis of studies involving dementia‐free individuals and found no significant pooled association between MetS and the incidence of overall dementia or AD in individuals with normal cognitive function or mild cognitive impairment (MCI). However, MetS was associated with a 1.37‐fold increased risk of developing pure VaD and a 2.69‐fold increased risk of dementia progression among patients with MCI. In another study, Lee et al. [21] followed 12,296,863 Korean adults without dementia (aged > 50 years) over a median period of 65 months. The metabolically healthy obese (MHO) group (BMI ≥ 25 kg/m^2^) showed a 15% and 13% reduced risk of incident dementia and AD, respectively, compared with the non‐obese group. However, no significant association was observed between the MHO group and incident VaD. The remaining four components of MetS, excluding obesity, were significantly associated with an increased risk of dementia, with stronger associations observed for VaD.

#### 3.1.2 Studies Including Participants With Dementia

A cross‐sectional study involving 162 patients with AD [18], with a mean age of 72.5 years, found that AD patients with MetS had significantly lower Mini‐Mental State Examination (MMSE) scores compared to those without MetS (16.06, 95% CI: 14.96–17.15 vs. 17.79, 95% CI: 17.05–18.53; *p* < 0.0001). In addition, the transcranial Doppler breath‐holding index, an indicator of cerebral hemodynamics, was also lower in the AD group with MetS. Wu et al. [23] conducted a 10‐year follow‐up study of 5963 adults enrolled in the Taiwan Biobank, including participants with dementia. An increasing number of MetS components at baseline was associated with a higher risk of developing MCI and dementia compared to the non‐MetS group. Among individual MetS components, hypertension (MCI: HR = 1.203, *p* < 0.001; dementia: HR = 1.345, *p* < 0.001), abdominal obesity (MCI: HR = 1.137, *p* = 0.006; dementia: HR = 1.442, *p* < 0.001), and low HDL‐C levels (MCI: HR = 1.149, *p* = 0.007; dementia: HR = 1.364, *p* < 0.001) were significantly associated with increased risk of both MCI and dementia.

#### 3.1.3 Variability in MetS and Dementia Risk

Fan et al. [19] conducted a retrospective cohort study involving 3458 dementia‐free adults aged 40–80 years. After a 10‐year follow‐up, participants whose MetS status worsened over time (baseline negative, follow‐up positive) had a 2.22‐fold increased risk of developing dementia compared to those who remained MetS‐free throughout the study (adjusted HR = 2.22, 95% CI: 1.31–3.72, *p* = 0.003). However, no significant association was observed in participants whose MetS either improved or remained stable over time. Similar results were observed among participants aged 65 years and older (adjusted HR = 2.00, 95% CI: 1.17–3.43, *p* = 0.012). In another study, Wu et al. [23] found that both participants whose MetS resolved after 10 years and those who continued to meet MetS criteria remained at a significantly higher risk of developing dementia compared to the non‐MetS group (HR = 1.361, *p* = 0.023; HR = 1.363, *p* = 0.003, respectively).

#### 3.1.4 Variability in the Definition of MetS Across Studies

Across the included studies, the diagnostic criteria for MetS were not consistent. Although most cohort and cross‐sectional studies used NCEP ATP III‐based criteria [16, 18, 19, 20, 21, 23], component definitions, particularly waist circumference cutoffs, varied according to the study population, whereas one study applied the International Diabetes Federation criteria, which requires central obesity as an essential component [14]. Among studies using NCEP ATP III‐based definitions, MetS was associated with incident MCI and progression from MCI to dementia [16], lower global cognitive performance [18], poorer performance in selected cognitive domains [20, 23], and an increased risk of dementia among participants with worsening MetS [19]. However, associations with dementia outcomes were not consistent: some studies reported a more pronounced association with VaD than with AD [13, 21], whereas another study found no significant difference in dementia conversion according to MetS status [20]. The study using the IDF criteria reported poorer attention or processing speed, executive function, and verbal fluency but did not evaluate incident dementia [14]. Studies additionally examining insulin resistance indices [22], metabolic biomarkers [15], individual metabolic risk factors [24, 26], or genetic signatures [17, 27] were interpreted separately from studies primarily evaluating clinically defined MetS. This heterogeneity in MetS definitions, study populations, and cognitive outcomes limited direct comparisons and may partly explain the inconsistent associations reported between MetS and dementia risk.

### 3.2 MetS and the Risk of MCI

Ng et al. [16] conducted a study involving dementia‐free adults aged over 55 years and found that individuals with MetS had a 1.46‐fold higher risk of developing MCI after 3.8 years of follow‐up compared to those without MetS. Among MetS components, participants with abdominal obesity, diabetes mellitus (DM), and dyslipidemia had 1.41, 2.84, and 1.48‐fold higher risks of developing MCI, respectively, compared to those without these conditions. Having three or more MetS components was associated with a 1.58‐fold increased risk of developing MCI compared to having fewer than three. Among individuals with MCI, those with MetS had a 4.25‐fold higher risk of progressing to dementia compared to those without MetS. DM increased the risk of MCI progression to dementia by 2.47‐fold, and having three or more MetS components was associated with a 4.92‐fold higher risk compared to having fewer than three.

### 3.3 Other Metabolic Markers and the Risk of Dementia

Pillai et al. [15] reported that a higher plasma TG/HDL‐C ratio was associated with a faster decline in Clinical Dementia Rating–Sum of Boxes (CDR‐SB) scores in both MCI and dementia groups. Higher plasma apolipoprotein A1 (ApoA1) levels were linked to a faster decline in MMSE and logical memory scores in the MCI group. Conversely, elevated cerebrospinal fluid (CSF) ApoA1 levels were associated with only a modest decline in MMSE scores in the MCI group. Hishikawa et al. [22] conducted a retrospective cohort study of 570 Japanese patients with AD (mean age 79.8 years), dividing them into groups based on abdominal obesity. The AD group with abdominal obesity showed worse changes in the Homeostasis Model Assessment of Insulin Resistance (HOMA‐IR), the reactive hyperemia index (a measure of vascular reactivity), and cerebral white matter compared to the non‐abdominal obesity group. Deng et al. [24] conducted a cross‐sectional study of 1781 Chinese adults aged 60 years or older, of whom 186 (10.44%) had dementia symptoms. Risk factors for dementia included rural residence, older age, living alone, obesity, hypertension, coronary artery disease, and depression. Protective factors included smoking, consumption of freshwater fish, moderate or regular exercise, high‐intensity labor, daily housework, outdoor physical activity, media use (e.g., television, radio, newspapers), and participation in social activities. Kim et al. [26] conducted a cross‐sectional study of 19,935 Korean adults aged over 60 years. Men and women with a history of stroke had 5.14 and 2.55‐fold higher risks of prevalent dementia, respectively, compared to those without stroke. For exercise and hypertension, both men and women showed a lower risk of dementia compared to those without exercise or hypertension.

### 3.4 Genes Related to MetS and Dementia

Zhang et al. [17] reported that the *APOE* gene was associated not only with dementia but also with MetS and DM. Li et al. [27] identified a gene co‐expression module using sequencing data to explore the genetic links between AD and MetS. Eight genes (*ARHGAP4, SNRPG, UQCRB, PSMA3, DPM1, MED6, RPL36AL*, *and RPS27A*) were identified as having diagnostic relevance for both MetS and AD.

### 3.5 MetS and Cognitive Function Domains

Exalto et al. [20] studied 86 adults with MMSE scores ≥ 22 (mean age 66.7 years), of whom 35 (41%) met MetS criteria. No significant differences were observed between MetS and non‐MetS groups in global cognition, brain MRI findings (degenerative or vascular abnormalities), or CSF amyloid and tau levels (all *p* ≥ 0.05). However, the MetS group showed poorer performance in executive function, attention/processing speed, and visuoconstruction (*z*‐scores, *p* < 0.05). Over a mean follow‐up of 3.4 years, dementia incidence was similar in MetS and non‐MetS groups (17% each; *p* = 0.45). Marseglia et al. [14] conducted a cross‐sectional study of 1131 dementia‐free 70‐year‐olds, in which the MetS group performed worse in attention/processing speed, executive function, and language fluency compared to the non‐MetS group. MetS participants with comorbid heart disease or arteriosclerosis had poorer cognitive function than those with only one of these conditions. Wu et al. [23] found that MetS was associated with a higher risk of low orientation and visuospatial scores on the MMSE. Among MetS components, hypertension and low HDL‐C were linked to low orientation, language, and visuospatial scores, while hyperglycemia was associated with low visuospatial scores. Participants who maintained MetS for 10 years had a higher risk of low orientation and visuospatial scores compared to the non‐MetS group. Those who were non‐MetS at baseline but developed MetS at follow‐up had an increased risk of lower language scores compared to persistent non‐MetS individuals. Kaplan–Meier curves demonstrated a higher cumulative risk of decline in orientation, language, and visuospatial ability in the MetS group compared to the non‐MetS group. No significant group differences were observed in other MMSE domains, including registration, concentration, and memory recall.

### 3.6 Variability and Heterogeneity Across Studies

The methodological quality of the included studies was generally moderate or higher, although the level of evidence varied according to study design, sample size, study population, and outcome assessment. There was also variation in the methods used for quality assessment. In particular, cross‐sectional and secondary data–based studies are limited in their ability to establish causality, and these differences in design may partly explain the inconsistent findings reported across studies on MetS and dementia.

## 4 Discussion

This review summarized evidence on the association between MetS and dementia risk across studies involving older populations. Among studies using clinical definitions of MetS, MetS was associated with MCI, progression from MCI to dementia, selected cognitive impairments, and dementia risk; however, the strength and direction of these associations varied according to the MetS definition, study design, and cognitive outcome. Hypertension, abdominal obesity, and low HDL‐C were among the components associated with adverse cognitive outcomes in some studies. However, these component‐level findings were inconsistent across studies. Therefore, the findings support MetS as a useful risk marker rather than identifying individual components as distinct targets for intervention.

### 4.1 Biological Mechanisms Linking MetS and Dementia

Hypertension is known to increase the risk of brain atrophy, stroke, and cerebral small vessel disease. It also contributes to arterial stiffness and accelerates β‐amyloid accumulation in the brain. Consequently, hypertension may induce vascular endothelial dysfunction, hypoxia, reduced cerebral blood flow, and impaired cholinergic neurotransmission, ultimately raising the risk of cognitive impairment [25].

Studies using abdominal MRI in AD patients have shown that greater abdominal fat deposition is associated with poorer cognitive function [28]. Similarly, higher waist circumference, subcutaneous fat, and visceral fat area have been identified as risk factors for cognitive decline in older adults [29]. One potential mechanism underlying the association between abdominal obesity and cognitive decline involves abnormal adipokine secretion. Adipokines influence cognitive function, vascular integrity, arteriosclerosis, and insulin resistance [29, 30]. Inflammatory cytokines from excess abdominal adipose tissue may further impair cognition [29]. Increased adipose tissue also elevates leptin secretion, while leptin resistance in obesity reduces blood–brain barrier permeability. This diminishes leptin's neuroprotective effects, including modulation of hippocampal synaptic plasticity and inhibition of β‐amyloid accumulation [31]. In addition, insulin resistance induced by abdominal obesity disrupts insulin signaling in the brain, contributing to cognitive decline [32].

**FIGURE 2:**
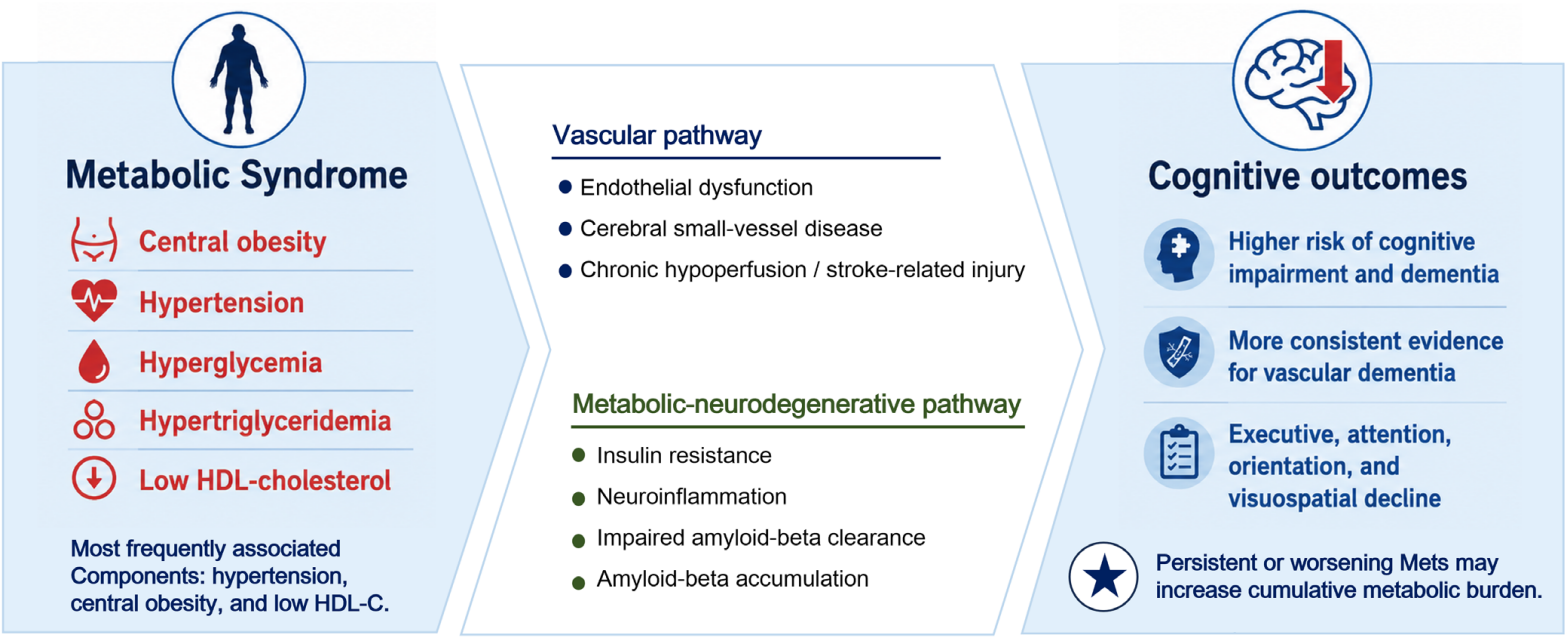
Proposed pathways from metabolic syndrome to cognitive outcomes.

Several studies have reported findings inconsistent with ours regarding the association between low HDL‐C levels and dementia risk [33, 34]. Although low HDL‐C has been associated with reduced gray matter volume in brain regions vulnerable to neurodegeneration [35], its relationship with dementia risk remains uncertain. Furthermore, adults with lower HDL‐C had a higher risk of memory loss compared to those with higher HDL‐C, regardless of *APOE* status [36]. Beyond quantitative levels, the functional properties of HDL‐C, such as its anti‐inflammatory and antioxidant capacity and its role in β‐amyloid clearance, may also contribute to neuroprotection [37, 38, 39]. Future studies should therefore examine not only HDL‐C levels but also particle quality and functionality, including reverse cholesterol transport and antioxidative effects, to clarify the potential mechanisms linking HDL‐C and dementia. Beyond the effects of individual components, dementia risk may increase as the number of MetS components increases [40]. The coexistence of multiple metabolic abnormalities may reflect a greater cumulative metabolic burden, contributing to systemic inflammation and neurovascular injury [41]. Therefore, MetS may serve as a composite risk marker for identifying individuals with a high overall metabolic burden, although its predictive value beyond individual components requires further investigation (Figure 2).

### 4.2 Heterogeneous Cognitive and Dementia Associations of MetS

In addition to global dementia risk, several studies highlighted domain‐specific associations between MetS and cognitive decline. Exalto et al. [20] and Marseglia et al. [14] reported that MetS was particularly linked to poorer performance in executive function, attention/processing speed, and language fluency, while Wu et al. [23] found associations with orientation and visuospatial abilities. These findings suggest that MetS may affect multiple cognitive domains through both vascular and neurodegenerative pathways [42]. MetS‐related hypertension, insulin resistance, dyslipidemia, endothelial dysfunction, and cerebral small vessel disease may impair frontal‐subcortical circuits through microvascular injury and chronic hypoperfusion [43, 44, 45, 46]. In cognitively normal individuals with pre‐existing amyloid positivity, MetS was also associated with accelerated amyloid‐β accumulation in the superior parietal and precuneus regions, with elevated fasting glucose and blood pressure showing individual associations with faster accumulation [11]. This posterior cortical involvement may partly explain the observed associations with orientation and visuospatial impairment. Differences in cognitive assessment methods may also contribute to variability across studies, as global screening tools such as the MMSE are less sensitive to executive and attentional deficits than detailed neuropsychological batteries.

All included studies involved elderly populations, and in these studies MetS was consistently associated with dementia risk. Given that the prevalence of MetS increases with age in Korean adults [5], these findings highlight the importance of MetS in dementia prevention among older individuals. However, as several reviewed studies also included younger and middle‐aged participants, the specific role of MetS in late life requires further investigation. Another important finding concerns the variability of MetS over time. Fan et al. [19] demonstrated that individuals whose MetS status worsened had a substantially increased risk of developing dementia, while those whose MetS improved or remained stable showed no significant reduction in risk. Similarly, Wu et al. [23] reported that both persistent MetS and resolution of MetS were associated with elevated dementia risk compared to non‐MetS individuals. These results suggest that early onset and long‐term persistence of MetS may have lasting effects on dementia risk, and highlight the importance of early detection and sustained management rather than delayed intervention [47, 48].

Some studies analyzed AD and VaD separately in addition to all‐cause dementia. One reported that AD was less strongly associated with certain MetS components, such as hypertension and DM, than VaD [49], whereas others found significant associations between MetS and AD [50, 51]. Hypertension and arteriosclerosis, which damage small cerebral vessels, are major contributors to VaD [52]. In AD, insulin resistance and chronic low‐grade inflammation may accelerate disease progression through increased β‐amyloid accumulation [53]. Although the mechanisms differ, evidence suggests that MetS and its components are related to both AD and VaD [54]. Currently, MetS management may be more directly beneficial in preventing VaD through vascular protection, while the association with AD may be more complex and indirect [55]. Additional studies are required to determine how individual MetS components contribute to the risk of each dementia subtype.

Of the 14 reviewed articles, two investigated genetic factors shared by MetS and dementia [17, 27]. Common gene expression patterns related to immune cell infiltration were identified [27], and an integrative analysis reported shared genes among MetS, dementia, and diabetes [17]. More recently, a multi‐omics study demonstrated genetic overlap between AD‐related gray matter atrophy and metabolic traits involving glucose and lipid metabolism [56]. These findings support shared molecular pathways linking metabolic dysregulation and neurodegeneration, although their clinical and predictive utility requires further validation.

### 4.3 Perspectives and Future Directions

As MetS and dementia are rapidly increasing public health challenges in aging societies, the association between these two conditions holds significant clinical and preventive importance [1, 4]. The high prevalence of MetS among individuals aged 65 and older suggests that it is a critical factor in the cognitive health of the elderly [5]. Based on the literature reviewed, MetS may be better understood as an accumulated metabolic burden resulting from long‐term metabolic abnormalities rather than a simple collection of independent risk factors [16, 40]. The risk of dementia and MCI increased with the number of MetS components, and MetS was also associated with a higher risk of progression from MCI to dementia [13, 16]. These findings suggest that MetS is a clinically relevant marker of cognitive risk.

The pathophysiological association between MetS and cognitive decline can be explained through several pathways. Inflammatory adipokine secretion and insulin resistance related to abdominal obesity are associated with β‐amyloid accumulation, impaired brain metabolism, and diminished neuroprotective mechanisms [30, 31, 32]. Concurrently, hypertension and dyslipidemia may contribute to white matter damage and impaired frontal‐subcortical circuit function by inducing microvascular injury, endothelial dysfunction, alterations in blood–brain barrier integrity, and chronic cerebral hypoperfusion [43, 44, 45, 46]. These multifaceted mechanisms may help explain the decline in executive function, attention, processing speed, and visuospatial function observed in patients with MetS [14, 20, 23]. This suggests that the impact of MetS on cognitive function likely results from the interaction between vascular and metabolic injuries rather than a single neurodegenerative pathway [42, 48].

Early detection and sustained management of MetS in clinical practice settings may represent important preventive strategies. Even when MetS status resolves, the risk of dementia may remain elevated compared with individuals who never met the MetS criteria. This implies that long‐term management beginning in midlife may be critical for preserving cognitive health [19, 23, 47, 48]. Future studies should adopt more comprehensive approaches, evaluating not only metabolic parameters but also the trajectory of MetS status and interactions with genetic predispositions such as *APOE* [17, 27]. In addition, long‐term follow‐up and intervention studies are necessary to confirm whether MetS management effectively prevents cognitive decline and dementia.

### 4.4 Strengths and Limitations

This review has several limitations. First, no randomized controlled trials (RCTs) were included; the available evidence was based on cohort and cross‐sectional studies, reflecting the nature of this research field. Second, the age range of participants was wide. Although all studies included older adults, it was difficult to clearly determine the specific risk of dementia attributable to MetS in late life, as some cohorts also involved younger adults. Third, the literature search focused primarily on the keywords “metabolic syndrome” and “dementia.” Although we included key studies addressing the full spectrum of cognitive impairment, including MCI, some studies indexed under terms such as “cognitive decline” or “MCI” may have been missed due to this search strategy. Fourth, the included studies varied in design, ranging from meta‐analyses to cross‐sectional studies. Although this provided a broader perspective, the heterogeneity in study design makes direct comparison difficult.

Despite these limitations, this review has several notable strengths. First, it focused on studies published within the last decade, ensuring that the most recent evidence was incorporated. Second, by including diverse study designs—prospective and retrospective cohorts, cross‐sectional analyses, meta‐analysis, and bioinformatics studies—this review provides a comprehensive overview of the current literature. Third, the review highlights important clinical and public health implications, suggesting that MetS management could play a role in preventing dementia, while also identifying research gaps—such as the role of genetic factors, HDL functionality, and variability in MetS status—that warrant further investigation.

## 5 Conclusions

In conclusion, this narrative review suggests that clinically defined MetS is associated with adverse cognitive outcomes, including incident MCI, progression from MCI to dementia, selected domain‐specific cognitive impairments, and an increased risk of dementia in some populations. However, the magnitude and consistency of these associations varied according to the diagnostic definition of MetS, study population, and cognitive outcome. Hypertension, abdominal obesity, and low HDL‐C emerged as potentially relevant components, but their associations were not uniform across studies and may not act as independent risk factors in all cases. Longitudinal changes in MetS status may also be important, particularly worsening or prolonged metabolic burden. Further prospective studies using standardized MetS definitions, repeated metabolic assessments, and clearly classified dementia outcomes are needed to clarify the contribution of MetS and its individual components to dementia risk.

## Funding

This study was supported by National Health Research Institutes (NHRI) under Grant Numbers NHRI‐11A1‐CG‐CO‐06–2225‐1, NHRI‐12A1‐CG‐CO‐06‐2225‐1, NHRI‐13A1‐CGCO‐06‐2225‐1, and NHRI‐14A1‐CG‐CO‐06‐2225‐1, as well as by the Kaohsiung Medical University Research Center Grant (KMU‐TC115B02), and Kaohsiung Medical University Gangshan Hospital (KMUGH‐DK(C)115001).

## Conflicts of Interest

The authors declare no conflicts of interest.

## Supporting information


**Table S1:** Quality assessment of included studies.

## Data Availability

Data sharing not applicable to this article as no datasets were generated or analysed during the current study.
