# Supplementary material for: Metabolic Syndrome and Dementia Risk in Older Adults: A Narrative Review
Source: Kaohsiung J Med Sci. 2026 Aug 19:e70276. Online ahead of print. doi: 10.1002/kjm2.70276 (PMC13487518; doi:10.1002/kjm2.70276)
Supplement: Supplementary file 1 [file KJM2-9999-e70276-s001.docx]

**Supplementary Table 1. Quality Assessment of Included Studies**

| **Study** | **Study design** | **Quality assessment method** | **Overall appraisal** |
| --- | --- | --- | --- |
| Li et al., 2022 | Bioinformatics research | No validated risk-of-bias tool applicable | Methodologically rigorous but limited to secondary data |
| Pillai et al. 2023 | Retrospective cohort | Newcastle–Ottawa Scale | 7/9 (High quality) |
| Atti et al. 2019 | Meta analysis | AMSTAR 2 | Moderate |
| Ng et al. 2016 | Prospective cohort | Newcastle–Ottawa Scale | 7/9 (High quality) |
| Zhang et al. 2017 | Bioinformatics research | No validated risk-of-bias tool applicable | Methodologically rigorous but limited to secondary data |
| Viticchi et al. 2015 | Cross-sectional | JBI Critical Appraisal Checklist | Moderate to High |
| Fan et al. 2017 | Retrospective cohort | Newcastle–Ottawa Scale | 8/9 (High quality) |
| Exalto et al. 2015 | Prospective cohort | Newcastle–Ottawa Scale | 6/9 (Moderate quality) |
| Lee et al. 2019 | Retrospective cohort | Newcastle–Ottawa Scale | 7/9 (High quality) |
| Hishikawa et al. 2016 | Retrospective cohort | JBI Critical Appraisal Checklist | Moderate |
| Marseglia et al. 2021 | Cross-sectional | JBI Critical Appraisal Checklist | Moderate |
| Wu et al. 2021 | Retrospective cohort | Newcastle–Ottawa Scale | 6/9 (Moderate quality) |
| Deng et al. 2018 | Cross-sectional | JBI Critical Appraisal Checklist | Moderate |
| Kim et al. 2017 | Cross-sectional | JBI Critical Appraisal Checklist | Moderate |

Abbreviations: JBI, Joanna Briggs Institute; AMSTAR 2, A Measurement Tool to Assess Systematic Reviews 2.
